# Supplementary material for: Broadband and fabrication-tolerant 3-dB couplers with topological valley edge modes
Source: Light Sci Appl. 2024 Jul 16;13:166. doi: 10.1038/s41377-024-01512-3 (PMC11250792; doi:10.1038/s41377-024-01512-3)
Supplement: Supplementary file 1 — Supplementary Information for Broadband and fabrication-tolerant 3-dB couplers with topological valley edge modes [file 41377_2024_1512_MOESM1_ESM.docx]

**Supplementary Information for**

**Broadband and fabrication-tolerant 3-dB couplers with topological valley edge modes**

Guo-Jing Tang^1,†^, Xiao-Dong Chen^1,†^ , Lu Sun^2,†^, Chao-Heng Guo^1^, Meng-Yu Li^1^, Zhong-Tao Tian^1^, Hou-Hong Chen^1^, Hong-Wei Wang^2^, Qi-Yao Sun^2^, Yun-Di Pan^2^ , Xin-Tao He^1,*^, Yi-Kai Su^2,*^ and Jian-Wen Dong^1,*^

*1, School of Physics & State Key Laboratory of Optoelectronic Materials and Technologies, Sun Yat-sen University, Guangzhou 510275, China.*

*2, State Key Laboratory of Advanced Optical Communication Systems and Networks, Department of Electronic Engineering, Shanghai Jiao Tong University, Shanghai 200240, China.*

^†^These authors contributed equally to this work

^*^Corresponding author: hext9@mail.sysu.edu.cn, yikaisu@sjtu.edu.cn, dongjwen@mail.sysu.edu.cn

This file includes:

**Appendix A: Topological property of valley photonic crystals**

**Appendix B: Group velocity and symmetry of edge states**

**Appendix C: Scattering matrix**

**Appendix D: Further discussion on robustness of topological coupler**

**Appendix E: Derivation and confirmation of interference process**

**Appendix F: Data analysis**

**Appendix G: The design and optimization of topological coupler**

**Appendix H: Data processing procedure of distance measurement**

**Appendix I: Comparison of various on-chip couplers**

# Appendix A: Topological property of valley photonic crystals

In this section, we calculate the band structure and Berry curvature distribution to confirm the nontrivial topological property of valley photonic crystals (VPCs). Fig. S1a illustrates the band structure of VPC used in topological coupler. A TE-like band gap spans from 1486 to 1585 nm. The topology of VPCs is characterized by the effective Hamiltonian $\delta H=v_{D}\left( \hat{\tau}_{z}\hat{\sigma}_{x}\delta k_{x}+\hat{\sigma}_{y}\delta k_{y} \right)+m\hat{\sigma}_{z}$, where $\hat{\sigma}_{i}$ and $\hat{\tau}_{i}$ are the Pauli matrices acting on sublattice and valley spaces, and *m* is coefficient of mass term whose sign will determine the valley Chern number by sgn[*C*_v_] = sgn[*m*]. Throughout this work, *C*_v1_ is the valley Chern number characterizing the first band of VPC1, and *C*_v2_ is for VPC2. To confirm the topological properties of the VPCs, we calculate the Berry curvature distributions of the first TE-like band. As shown in Fig. S1b, the Berry curvature distributions for VPC1 are localized around K and K’ points in reciprocal space. Similar results can also be seen for VPC2 but have opposite signs to VPC1. By integrating the Berry curvature in the half Brillouin zone around K and K’ valleys, we can derive the valley index *C*_K_ and *C*_K’_ and calculate the valley Chern numbers by *C*_v_ = *C*_K_ - *C*_K’_. We can find that *C*_v1_ < 0, *C*_v2_ > 0. According to the relation between *C*_v_ and *m*, we can derive that the sign of mass term coefficient for VPC1 (VPC2) is negative (positive), i.e. *m*_1_ < 0 (*m*_2_ < 0).


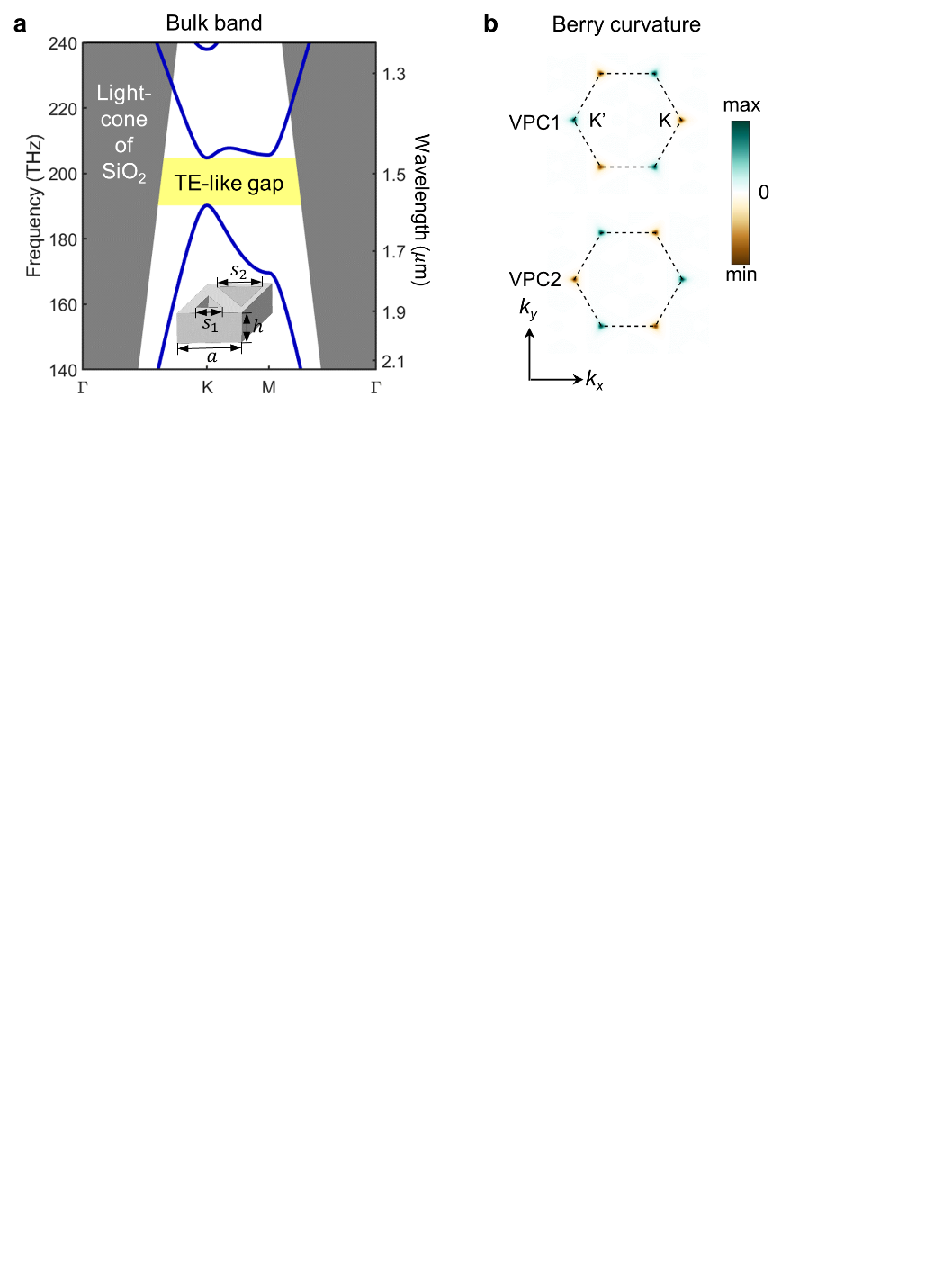


**Fig. S1 Bulk band and Berry curvature distributions of VPCs.** **a**, Bulk band of TE-like modes of VPC slabs. The band gap is highlighted in yellow. The lightcone of SiO_2_ is colored in gray. **b**, Berry curvature distributions of the first bulk band. The dashed hexagon is the first Brillouin zone.

# Appendix B: Group velocity and symmetry of edge states

In this section, we elucidate the impact of valley-Hall topology and mirror symmetry on the properties of edge states. Firstly, we deduce the propagation direction and symmetry of edge states around K and K’ valleys based on the effective Hamiltonian of VPCs. Subsequently, we confirm the theoretical conclusions with simulation results.


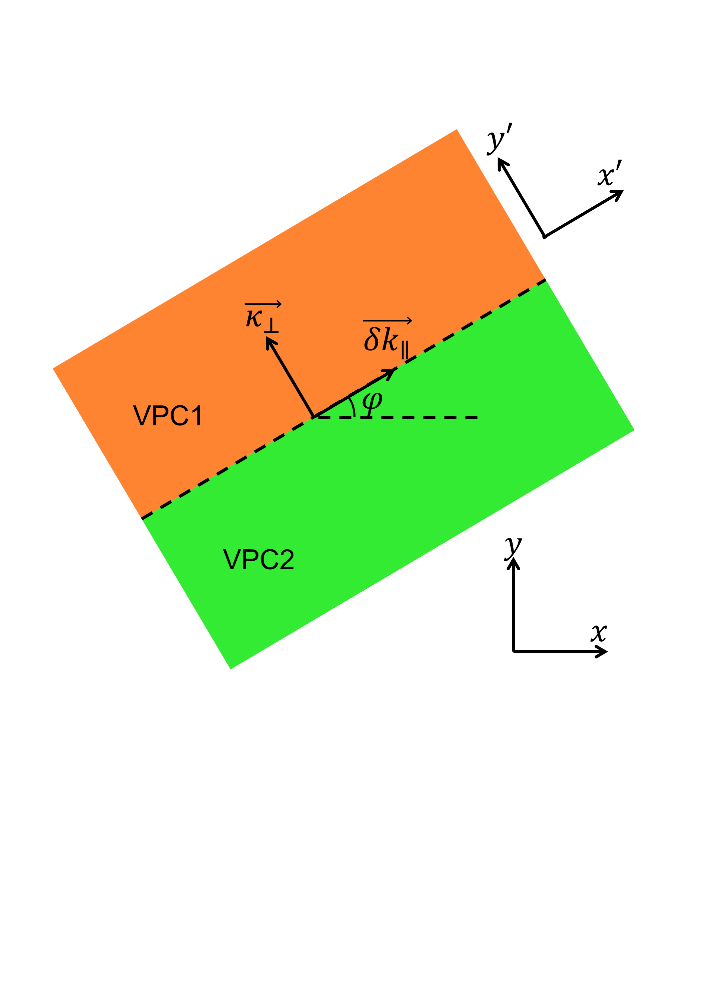


**Fig. S2 Illustration of an edge between VPCs with opposite valley Chern numbers.** The relations between $\vec{k_{\parallel}}$, $\vec{\kappa_{\perp}}$ and φ are shown clearly. The VPC1 and VPC2 are colored in orange and green, respectively.

We begin from the photonic effective Hamiltonian of VPCs around K and K’ valleys:

$\delta H=v_{D}\left( \hat{\tau}_{z}\hat{\sigma}_{x}\delta k_{x}+\hat{\sigma}_{y}\delta k_{y} \right)+m\hat{\sigma}_{z}$ (S1)

where $v_{D}$ is the group velocity for the bulk states near Dirac point when the band gap is closed. $m$is the mass term and determines the valley Chern number of VPCs. $\hat{\sigma}_{i}$ and $\hat{\tau}_{i}$ are the Pauli matrices acting on sublattice and valley space. When we focus on the edge states localized at interface I_21_ around K valley, the effective Hamiltonian is

$\delta H=\left\{ \begin{aligned} v_{D}\left[ \hat{\sigma}_{x}\left( \delta k_{x}'\cos\varphi-\delta k_{y}'\sin\varphi\right)+\hat{\sigma}_{y}\left( \delta k_{x}'\sin\varphi+\delta k_{y}'\cos\varphi\right) \right]-m_{b}\hat{\sigma}_{z} \left( y'>0 \right) \\ v_{D}\left[ \hat{\sigma}_{x}\left( \delta k_{x}'\cos\varphi-\delta k_{y}'\sin\varphi\right)+\hat{\sigma}_{y}\left( \delta k_{x}'\sin\varphi+\delta k_{y}'\cos\varphi\right) \right]+m_{b}\hat{\sigma}_{z} \left( y'<0 \right) \end{aligned} \right.$ (S2)

and the edge states has expression

$H_{z}=\left\{ \begin{aligned} \left( a_{u}H_{R}+b_{u}H_{L} \right)e^{i\delta\vec{k}\cdot\vec{r}} \left( y'>0 \right) \\ \left( a_{l}H_{R}+b_{l}H_{L} \right)e^{i\delta\vec{k}\cdot\vec{r}} \left( y'<0 \right) \end{aligned} \right.$ (S3)

where $H_{R}$ and $H_{L}$ are the states that $H_{z}$ fields have phase vortexes of different directions.

Because edge state decay exponentially alone the direction of $\vec{\kappa_{\perp}}$, we set

$\delta k_{x}^{'}=\delta k_{\parallel}$, $\delta k_{y}^{'}=\left\{ \begin{aligned} i\kappa_{\perp} \left( y'>0 \right) \\ -i\kappa_{\perp} \left( y'<0 \right) \end{aligned} \right.$

where $\kappa_{\perp}$ and $\delta k_{\parallel}$ are real and $\kappa_{\perp}>0$. By solving the eigenequation $\delta H H_{z}=\delta\omega H_{z}$, we can get

$\kappa_{\perp}=\sqrt{{\delta k_{\parallel}}^{2}-\frac{{\delta\omega}^{2}-{m_{b}}^{2}}{{v_{D}}^{2}}}$ (S4)

To get eigenstate we substitute the expression of $H_{z}$ into the effective Hamiltonian and have

$\frac{a_{u}}{b_{u}}=\frac{v_{D}\left( \delta k_{\parallel}+\kappa_{\perp} \right)e^{-i\varphi}}{\delta\omega+m_{b}} \left( y'>0 \right)$

$\frac{a_{l}}{b_{l}}=\frac{v_{D}\left( \delta k_{\parallel}-\kappa_{\perp} \right)e^{-i\varphi}}{\delta\omega-m_{b}} \left( y'<0 \right)$

Applying the boundary condition of electromagnetic field and considering the orthogonality of $H_{L}$ and $H_{R}$ states, we can derive that $a_{u}=a_{l} , b_{u}=b_{l}$. Combining these relations with the condition $-m_{b}<\delta\omega<m_{b}$, we can obtain the dispersion of edge states near Dirac frequency at K point

$\delta\omega=v_{D} \delta k_{\parallel}$ (S5)

and the ratio of $a_{u}$ ($a_{l}$) and $b_{u}$ ($b_{l}$) can be derivated

$\frac{a_{u}}{b_{u}}=\frac{a_{l}}{b_{l}}=-e^{-i\varphi}$ (S6)

For K’ point and the cases of $m_{b}<0$ (interface I_12_), we can get results by similar method. In summary, the field distributions and the decay coefficients are expressed as

$H_{z}=\left( H_{R}+sgn\left[ m_{b} \right]e^{i\varphi}H_{L} \right)\exp\left( i\vec{\delta k_{\parallel}}\cdot\vec{r}-\left| \vec{\kappa_{\perp}}\cdot\vec{r} \right| \right)$ (S7)

The group velocities $v_{g}=\frac{\delta\omega}{\delta k_{\parallel}}$, which determines the propagation direction of edge states, are derived

$v_{g}=sgn\left[ m_{b} \right]\hat{\tau}_{z}v_{D}$ (S8)

Because sgn[*C*_vb_] = sgn[*m*_b_], we can derive the sign of group velocity in main text.

Then we consider the symmetry of edge states. In the case of $\varphi=0$, the edge states localized at the interface I_21_ have the form of

$H_{z21}=\left( H_{R}+H_{L} \right)e^{i\delta k_{x}x-\kappa_{\perp}\left| y \right|}$ (S9)

and the edge states localized at the interface I_12_ have the form of

$H_{z12}=\left( H_{R}-H_{L} \right)e^{i\delta k_{x}x-\kappa_{\perp}\left| y \right|}$ (S10)

Due to the mirror symmetry of the zigzag interfaces, the distributions of edge states also serve as the eigenfields of the mirror operation. The field distributions of $H_{R}$ and $H_{L}$ form a pair of mirror symmetry partners, given by $\mathcal{M}\left( H_{R} \right)=H_{L}$, $\mathcal{M}\left( H_{L} \right)=H_{R}$, where $\mathcal{M}$ represent mirror symmetry operation. By utilizing these mirror symmetry relationships, we can derive the following result:

$\mathcal{M}\left( H_{z21} \right)=H_{z21}$, $\mathcal{M}\left( H_{z12} \right)=-H_{z12}$ (S11)

When we consider the sign of $m_{b}$, the $H_{z}$ field symmetry of edge states can be expressed as

$\mathcal{M}\left( H_{z} \right)=sgn\left[ m_{b} \right]H_{z}$ (S12)

Therefore, the $H_{z}$ fields of edge states localized at interface I_21_ has even symmetry, while the $H_{z}$ fields of edge states localized at interface I_12_ has odd symmetry. Because *H* is a pseudo-vector, the symmetry of edge state and $H_{z}$ field are opposite.

To confirm the theoretical results, we calculate the dispersion and show the *H_z_* fields of edge states. Eq. S8 indicates the group velocity of edge states supported by Edge21 (Edge12) with *m*_b_ < 0 (*m*_b_ > 0) is positive (negative) at K valley while is negative (positive) at K’ valley. According to Eq. S12, edge states with symmetric (anti-symmetric) *H_z_* field distribution are supported by the Edge21 (Edge12). All these conclusions are confirmed by the simulation results in Fig. S3.


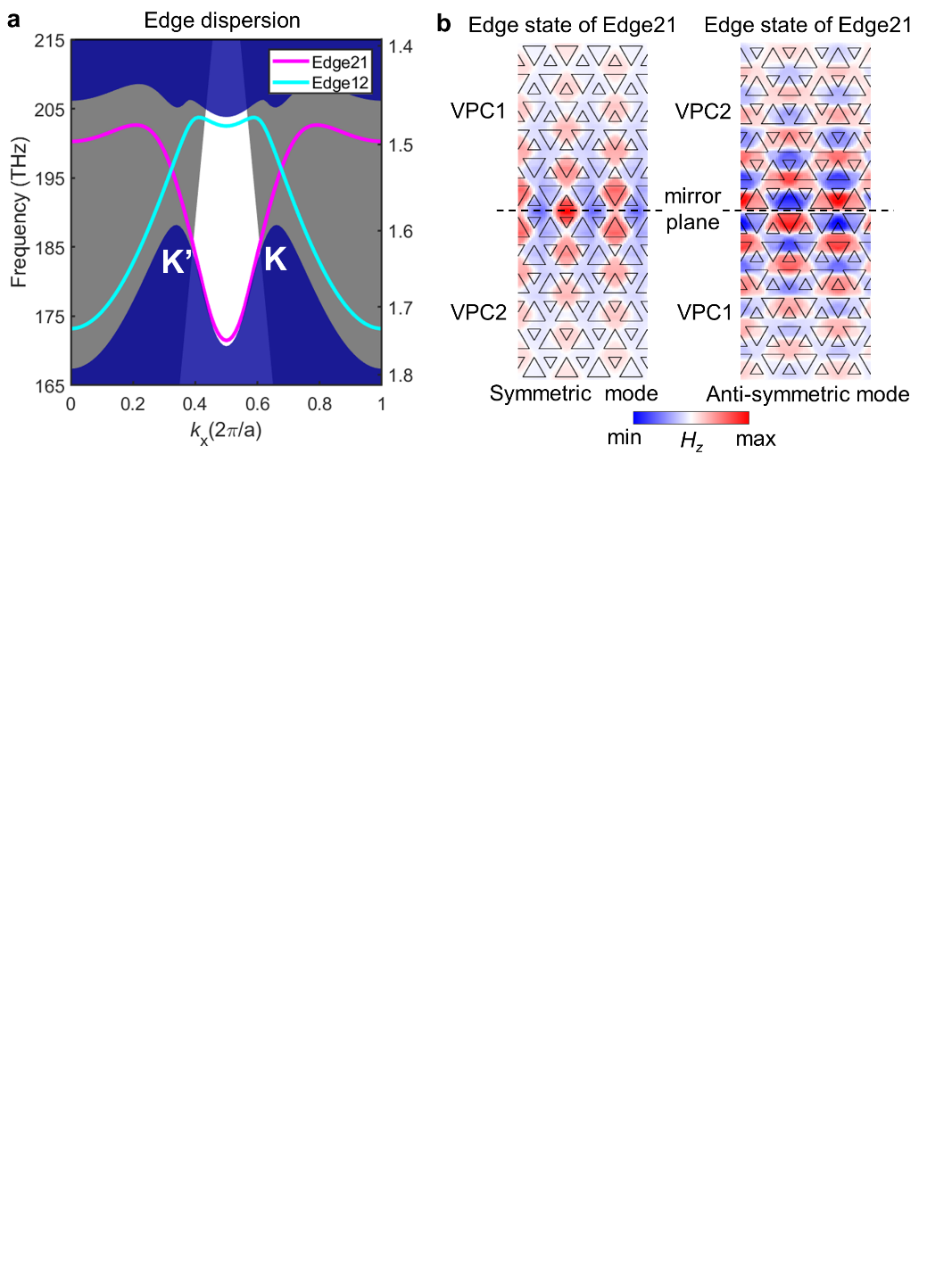


**Fig. S3 Dispersions and *H_z_*field distributions of edge states. a**, Dispersions of edge states of Edge21 (magenta) and Edge12 (cyan). The projected bulk band is colored in dark bule. The lightcone of SiO_2_ is colored in gray. **b**, *H_z_* field distributions of edge states of Edge21 (left) and Edge12 (right) at K point. The dash lines denote the mirror plane of VPC structures.

# Appendix C: Scattering matrix

In this section, we will derive the scattering matrix of the topological 3-dB coupler. The inter-valley scattering suppression leads to the vanishing of certain elements in the scattering matrix. When light is input from port 1 or port 2, light will only output to port 3 and port 4. Conversely, when light is input from port 3 or port 4, it will only output to port 1 and port 2. Consequently, the diagonal blocks of scattering matrix can be eliminated and the off-diagonal blocks describe the transmitting relations between two sets of ports. The scattering matrix takes the following form:

$\left( \begin{aligned} \begin{matrix} \begin{matrix} H_{1}^{\mathrm{out}} \\ H_{2}^{\mathrm{out}} \end{matrix} \\ H_{3}^{\mathrm{out}} \end{matrix} \\ H_{4}^{\mathrm{out}} \end{aligned} \right)=\left( \begin{matrix} 0 & \begin{matrix} s_{13} & s_{14} \\ s_{23} & s_{24} \end{matrix} \\ \begin{matrix} s_{31} & s_{32} \\ s_{41} & s_{42} \end{matrix} & 0 \end{matrix} \right)\left( \begin{aligned} \begin{matrix} \begin{matrix} H_{1}^{\mathrm{in}} \\ H_{2}^{\mathrm{in}} \end{matrix} \\ H_{3}^{\mathrm{in}} \end{matrix} \\ H_{4}^{\mathrm{in}} \end{aligned} \right)$ (S13)

where $H_{i}^{in/out}$represent the amplitude of the input/output edge states at port *i*.

The mirror symmetry of topological 3-dB coupler ensures the equal proportion of splitting. Due to the structural mirror symmetry, the scattering matrix remains unchanged under mirror operation, i.e. $\mathcal{M}\left( S \right)=S$.

When we operate mirror symmetry on the field distribution for the cases of inputting from port 1 (port 2), the *H_z_* field has symmetric (anti-symmetric) distribution,

$\mathcal{M}\left( H_{z, port1} \right)=H_{z, port1}$ (S14a)

$\mathcal{M}\left( H_{z, port2} \right)=-H_{z, port2}$ (S14b)

The second subscript indicates the input port. As shown in Fig. S4, we have

$\mathcal{M}\left( H_{1, port1}^{\mathrm{in}} \right)=H_{1, port1}^{\mathrm{in}}$, $\mathcal{M}\left( H_{3/4, port1}^{\mathrm{out}} \right)=H_{3/4, port1}^{\mathrm{out}}$ (S15a)

$\mathcal{M}\left( H_{2, port2}^{\mathrm{in}} \right)=-H_{2, port2}^{\mathrm{in}}$, $\mathcal{M}\left( H_{3/4, port2}^{\mathrm{out}} \right)=-H_{3/4, port2}^{\mathrm{out}}$ (S15b)

When we operate mirror symmetry on the whole structure, the mirror operation is equivalent to exchange port 3 and port 4. We have

$\mathcal{M}\left( H_{3/4, port1}^{\mathrm{out}} \right)=H_{4/3, port1}^{\mathrm{out}}$ (S16a)

$\mathcal{M}\left( H_{3/4, port2}^{out} \right)=H_{4/3, port2}^{\mathrm{out}}$ (S16b)

So, we can derive

$H_{3/4, port1}=H_{4/3, port1}$ (S17a)

$H_{3/4, port2}=-H_{4/3, port2}$ (S17b)

which indicates $s_{31}=s_{41}$, $s_{32}=-s_{42}$. Considering the energy conservation condition, we can derive the scattering matrix with the form:

$\left( \begin{aligned} \begin{matrix} \begin{matrix} H_{1}^{\mathrm{out}} \\ H_{2}^{\mathrm{out}} \end{matrix} \\ H_{3}^{\mathrm{out}} \end{matrix} \\ H_{4}^{\mathrm{out}} \end{aligned} \right)=\left( \begin{matrix} 0 & \begin{matrix} s_{13} & s_{14} \\ s_{23} & s_{24} \end{matrix} \\ \begin{matrix} \frac{\sqrt{2}}{2} & \frac{\sqrt{2}}{2} \\ \frac{\sqrt{2}}{2} & -\frac{\sqrt{2}}{2} \end{matrix} & 0 \end{matrix} \right)\left( \begin{aligned} \begin{matrix} \begin{matrix} H_{1}^{\mathrm{in}} \\ H_{2}^{\mathrm{in}} \end{matrix} \\ H_{3}^{\mathrm{in}} \end{matrix} \\ H_{4}^{\mathrm{in}} \end{aligned} \right)$ (S18)


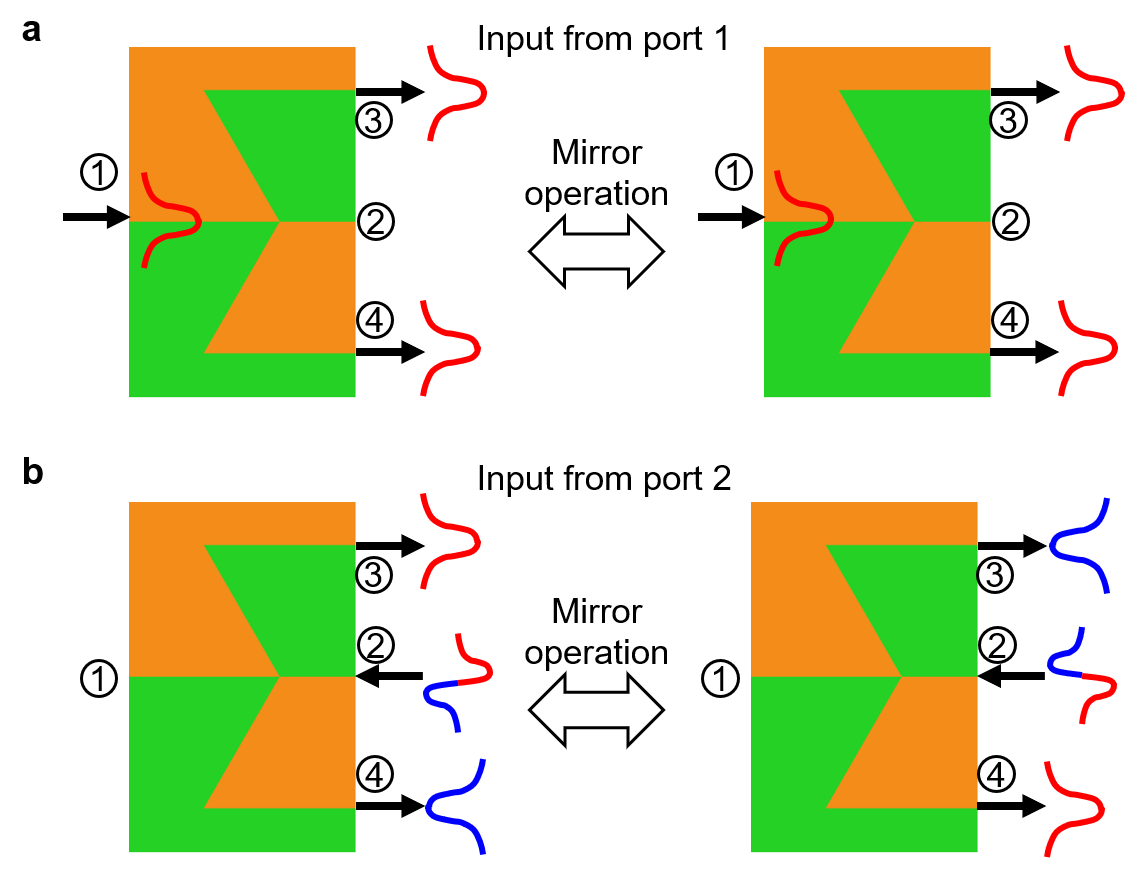


**Fig. S4 Theoretical analysis of *H_z_* field distributions for inputting from port1 and port 2.** The cases of inputting from port1 (a) and port 2 (b) are analyzed. The Gaussian-shape curves represent edge states with symmetric *H_z_* distribution, which appear at port 1, port 3 and port 4. The S-shape curves represent edge states with anti-symmetric *H_z_* distribution, which appear at port 2. Red and blue represent opposite amplitude, i.e. phase difference of π.

To confirm the theoretical results, we perform the simulations with light input from port 1 and port 2. As shown in Fig. S5, the field remains unchanged when we apply mirror symmetry to the input field for the case of inputting from port 1. While for the case of inputting from port 2, the *H_z_* filed will undergoes a phase reversal under mirror symmetry operation. The simulation results coincide with theoretical analysis.


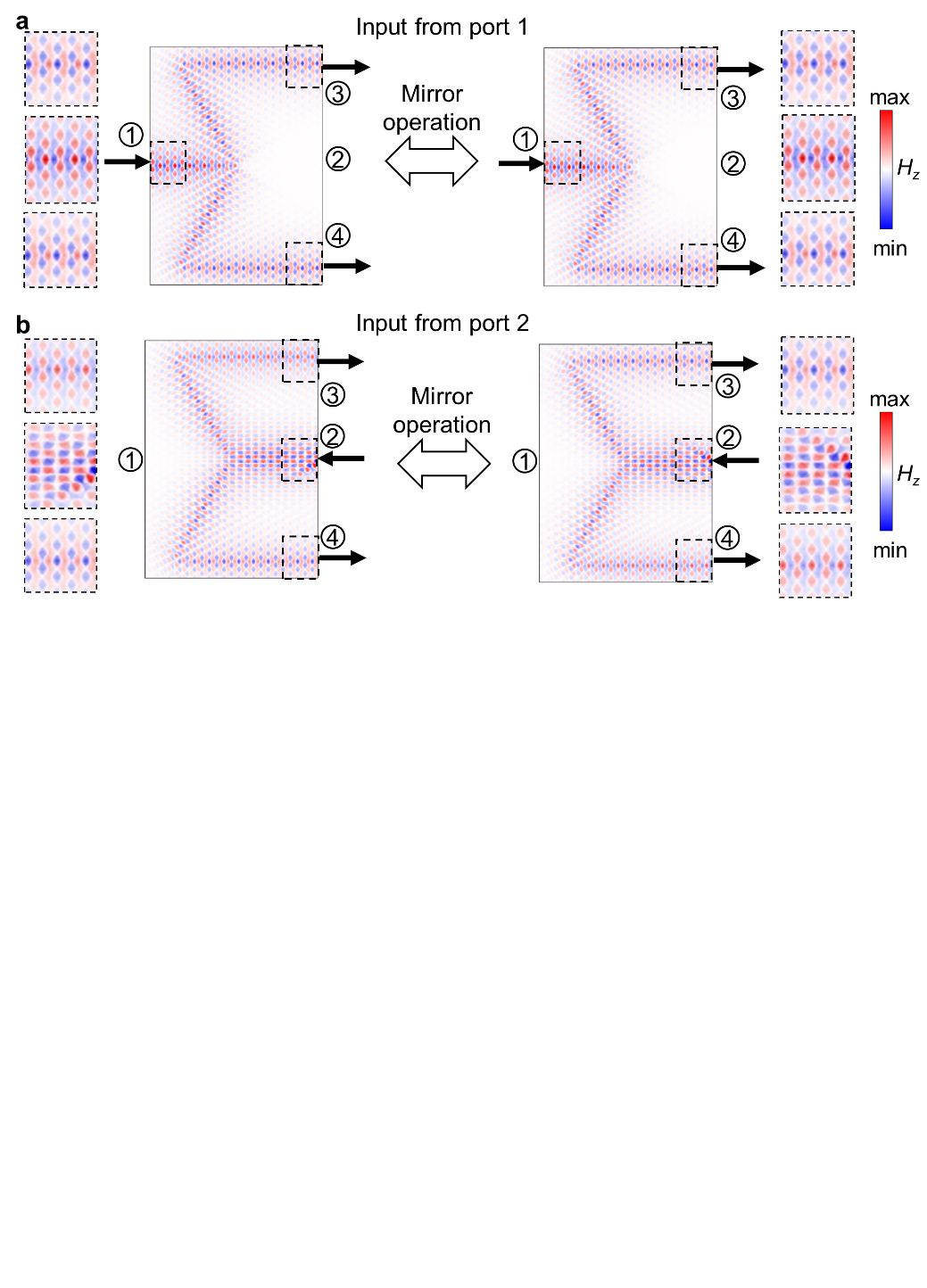


**Fig. S5 Simulation results of *H_z_* field distribution for input from port1 and port 2.** To clearly show the effect of mirror operation, the *H_z_* field distributions of input and output ports are zoomed in.

After analyzing the splitting with symmetric excitation (i.e., from port 1 and port 2), we will discuss the splitting process in the case of asymmetric excitation (i.e., from port 3 and port 4). When light is input from port 3 or port 4, the splitting ratio cannot be derived directly with symmetry, as the excited modes are not symmetric with respect to the mirror plane of the entire structure. However, we notice that the field distributions when inputting from port 3 and port 4 are mirror symmetry partners. The mirror operation is equivalent to exchanging port 3 and port 4. Thus, the field distributions of the transmitted waves excited from port 3 and port 4 can be transformed into each other by applying a mirror operation and adding a phase shift arising from the phase difference between the input sources. Because the edge states output to port 1 (port 2) are symmetric (anti-symmetric) modes, as depicted in Fig. S6, we have

$\mathcal{M}\left( H_{1, port3}^{\mathrm{out}} \right)=H_{1, port4}^{\mathrm{out}}$ (S19a)

$\mathcal{M}\left( H_{2, port3}^{\mathrm{out}} \right)=-H_{2, port4}^{\mathrm{out}}$ (S19b)

It indicates that $s_{13}=s_{14}$, $s_{23}=-s_{24}$.


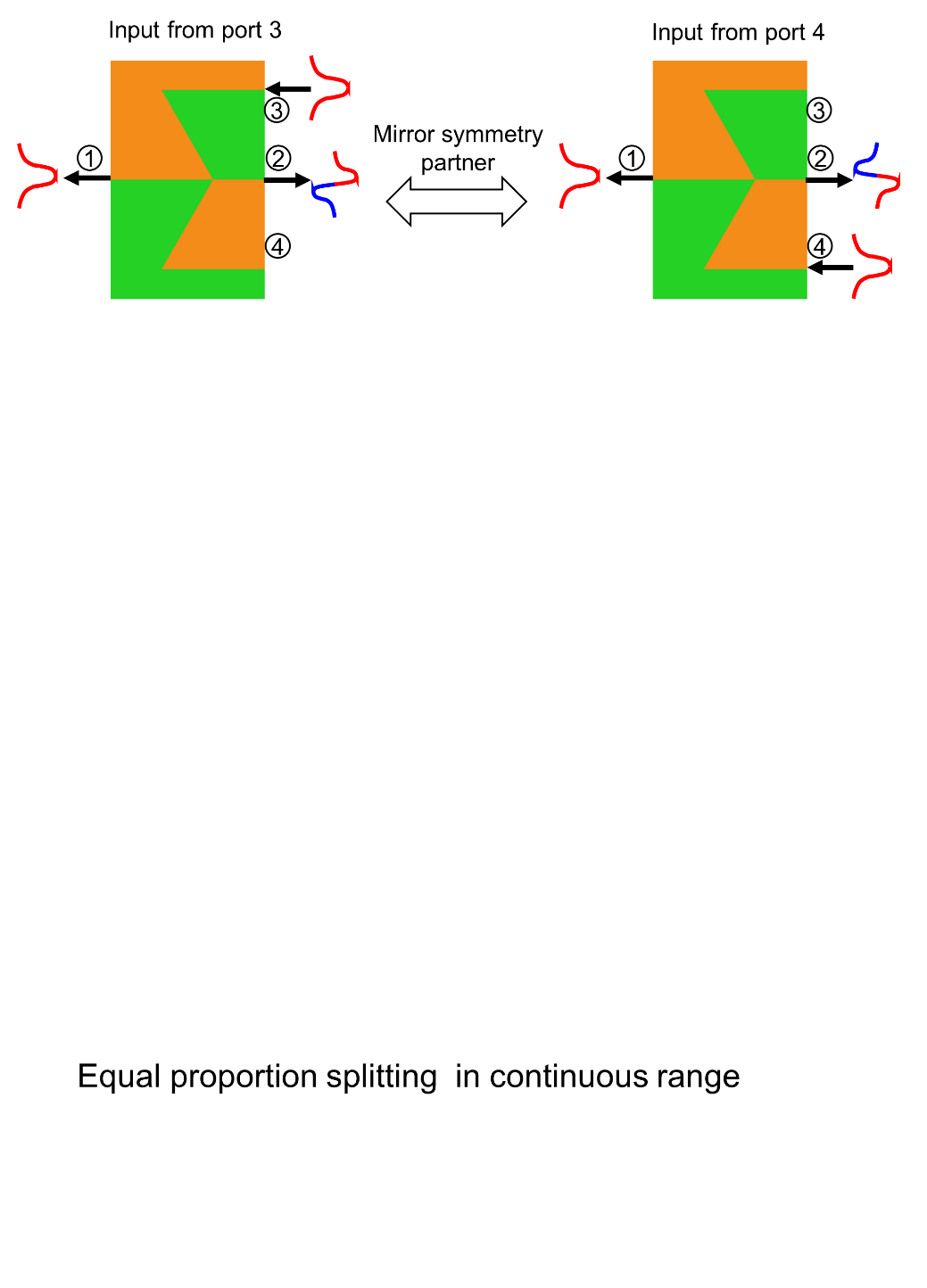


**Fig. S6 Theoretical analysis of *H_z_* field distributions for inputting from port1 and port 2.** The cases of inputting from port 3 (left) and port 4 (right) are analyzed. The two cases are a pair of mirror symmetry partner.

To derive the exact values of the top-right block of scattering matrix, we consider the superpositions of the cases of inputting from port 3 and port 4. When we add the field distributions of these two cases, the output field at port 2 is vanished because they are a pair of mirror symmetric partners and the waveguide to port 2 supports edge modes with anti-symmetric distributions. This superposition is just the time reversal case of inputting from port 1. When we subtract these two field distributions, we obtain the time reversal case of inputting from port 2. Taking the energy conservation condition into consideration, we can derive that $\left| s_{13} \right|^{2}+\left| s_{14} \right|^{2}=\left| s_{23} \right|^{2}+\left| s_{24} \right|^{2}=1$. Therefore, the parameters are derived as $\left| s_{13} \right|=\left| s_{14} \right|$=$\left| s_{23} \right|=\left| s_{24} \right|=\frac{\sqrt{2}}{2}$. We finally derive the scattering matrix:

$S=\frac{\sqrt{2}}{2}\left( \begin{matrix} 0 & \begin{matrix} 1 & 1 \\ 1 & -1 \end{matrix} \\ \begin{matrix} 1 & 1 \\ 1 & -1 \end{matrix} & 0 \end{matrix} \right)$ (S20)

To confirm our theoretical results, we perform the transport simulations with light input from port 3 and port 4. As shown in Fig. S7, these two cases are mirror symmetry partners. When comparing them, we can observe that the field of port 1 remain unchanged, while the field of port 2 undergoes a phase reversal. The simulation results coincide with theoretical analysis.


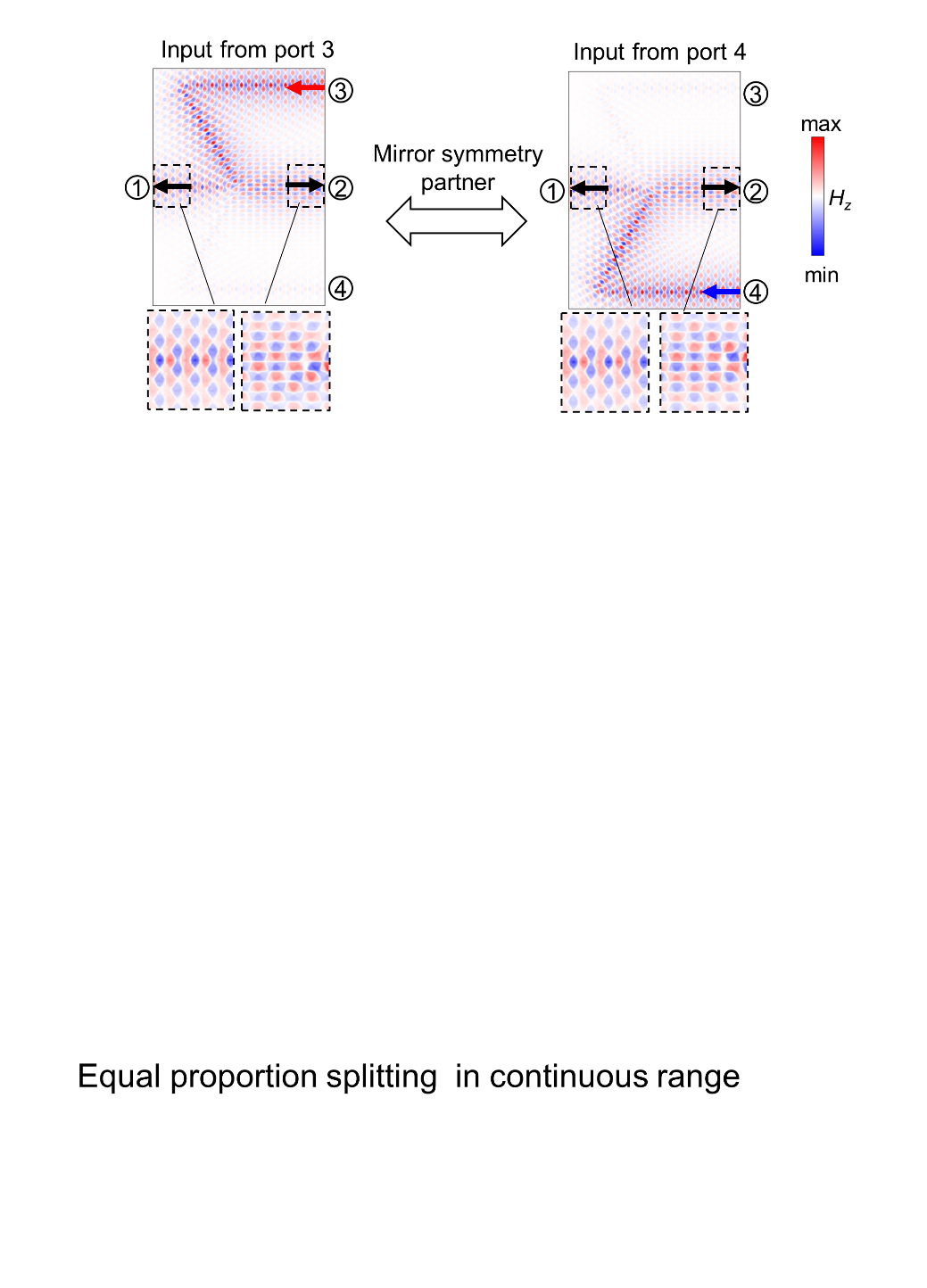


**Fig. S7 Simulation results of *H_z_* field distribution for input from port1 and port 2.** To clearly show the effect of mirror operation, the *H_z_* field distributions of output ports are zoomed in.

# Appendix D: Further discussion on robustness of topological coupler

In this section, we present the simulation results of the error analysis of topological coupler and conventional DC. We then discuss the robustness of couplers against temperature variation. Finally, the error analysis of cascaded couplers is performed. Temperature drift and cascade are commonly encountered in photonic integrated circuit systems featuring thermo-optical devices.


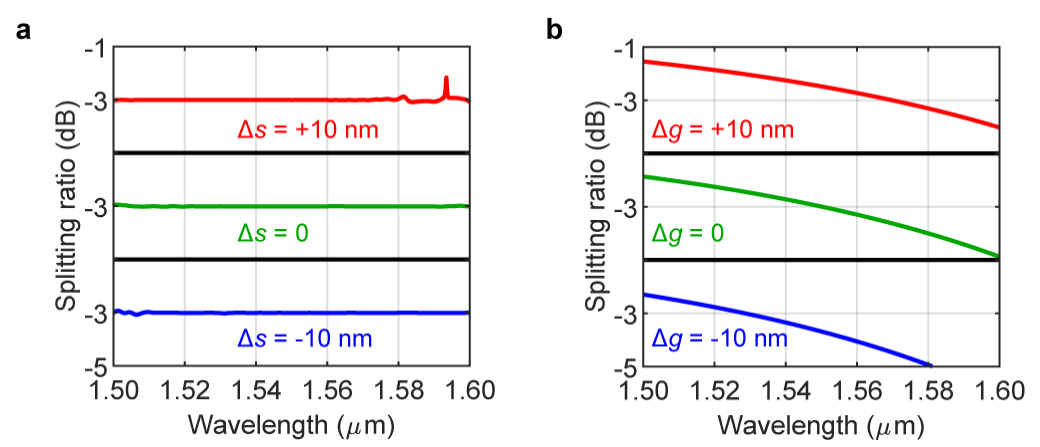


**Fig. S8 Error analysis of single couplers in simulations.** Simulation splitting ratio spectra of (**a**) topological coupler and (**b**) conventional DC with different errors when inputting light from port 1.

Similar to the experimental analysis presented in the main text of Fig. 3, we also perform the simulation results of error analysis in Fig. S8. The shapes of the simulation spectra of topological coupler remain flat for a broadband range that exactly reach at the value of 3 dB, while the simulated ratio spectra of conventional DC exhibit obviously oblique slopes and the 3-dB splitting only occurs at a single wavelength. Note that the simulation splitting ratio spectra appear smoother, since simulations are not affected by the unavoidable measuring errors that can occur in experiments.

We compare the temperature stability of topological coupler and conventional directional coupler (DC) in simulation. When the temperature variation is introduced, the dominant effect on integrated devices is the changing of the refractive index of silicon [1]. Considered temperatures of $T=100K, 400K, 700K$, we can have the refractive indices of silicon as *n*_Si_ = 3.4467, 3.4966 and 3.5648, respectively. The change of refractive index leads to shifts in the band gap and 3-dB splitting range. Benefiting from the broad bandwidth of the topological 3-dB coupler, there is still overlapped wavelength range of 3-dB splitting ratio. As shown in Fig. S9a, the splitting ratio remains within (3 ± 0.1) dB in 63 nm (1527 ~ 1590 nm) even when considering a ±300 $K$ temperature variation. In contrast, the analysis of a conventional DC is shown in Fig. S9b. The 3-dB splitting occurs only at single wavelength, which shifts under different temperatures.


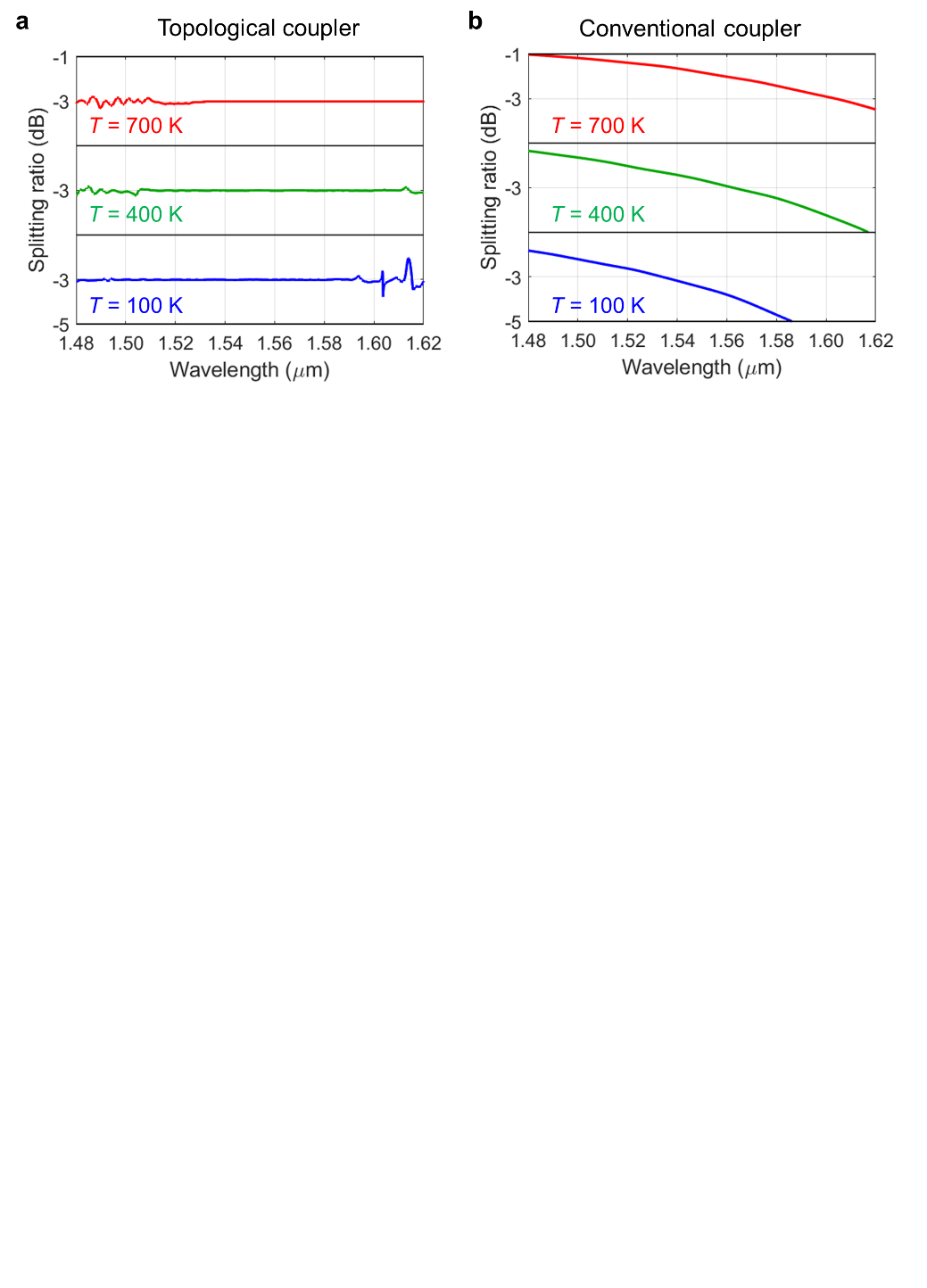


**Fig. S9 Temperature characteristic analysis.** Splitting ratio spectra of (**a**) topological coupler and (**b**) conventional DC in the case of inputting light from port 1. The cases of $T=100K, 400K, 700K$ are shown.

Next, we discuss the robustness against dimensional error in cascaded couplers. We construct two 2×4 splitting structures in Figs. S10a and S10d containing three 3-dB couplers. The dimensional error is introduced in the same manner as in the case of single coupler (see Fig. 3 in the main text). Figure S10b shows the cross-section profile of the electromagnetic field intensity at the output waveguides of topological cascaded coupler. We can see the output powers of four ports at λ = 1550 nm remain almost the same intensity, even with dimensional error of Δs = ±10 nm. The splitting ratio spectra of port 4 are also shown in Fig. S10c, indicating that the splitting ratio remains flat and within (-6 ± 0.6) dB from 1535 nm to 1577 nm for these cases. In comparison, the output EM field intensity of cascaded DC have significant difference, even though the case of Δg = 0 exhibits the same intensity (Fig. S10e). As shown in Fig. S10f, the splitting ratio spectra of port 4 in cascaded conventional DC exhibit obvious oblique slopes, which are even larger than the case of single coupler. Consequently, the cascading of couplers will increase the sensitivity of conventional DC against dimensional error, while topological couplers remain robust against dimensional error even in the cascade case.


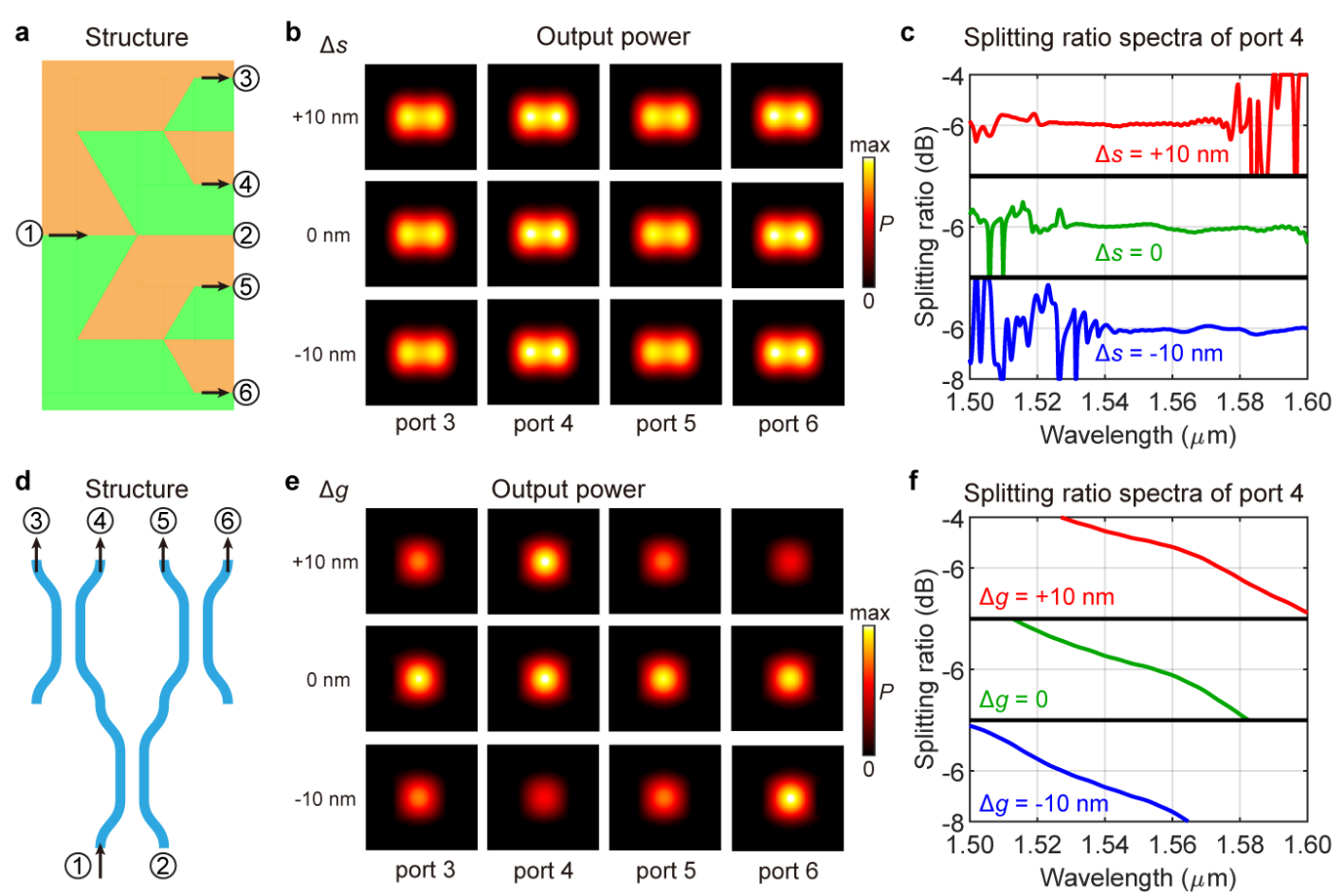


**Fig. S10 Tolerance analysis of the cascaded couplers. a** and **d**, Schematic of cascaded couplers constructed with three single (**a**) topological couplers and (**d**) conventional DCs. **b** and **e**, Power field distributions of the waveguides at four output ports in (**b**) topological and (**e**) conventional cascaded coupler. The light source is the mode source input from port 1. The wavelength of source is 1550 nm. **c** and **f**, Splitting ratio spectra of port 4 in simulations for (**b**) topological and (**d**) conventional cascaded couplers with different errors when inputting light from port 1.

Finally, we discuss the robustness against non-uniform dimensional errors. We introduce non-uniform errors on different holes of the topological coupler. The dimensional errors are in the range of $-10 \mathrm{nm} \leq\Delta s \leq10 \mathrm{nm}$. Random fluctuations will break down mirror symmetry of structure, leading small variation on splitting ratio. We perform the full-wave simulations with random dimensional errors for 100 times. According to the results in Fig. S11, the splitting ratio still keep within (3 ± 0.32) dB in a bandwidth of 55 nm, which has little influence on the splitting performance.


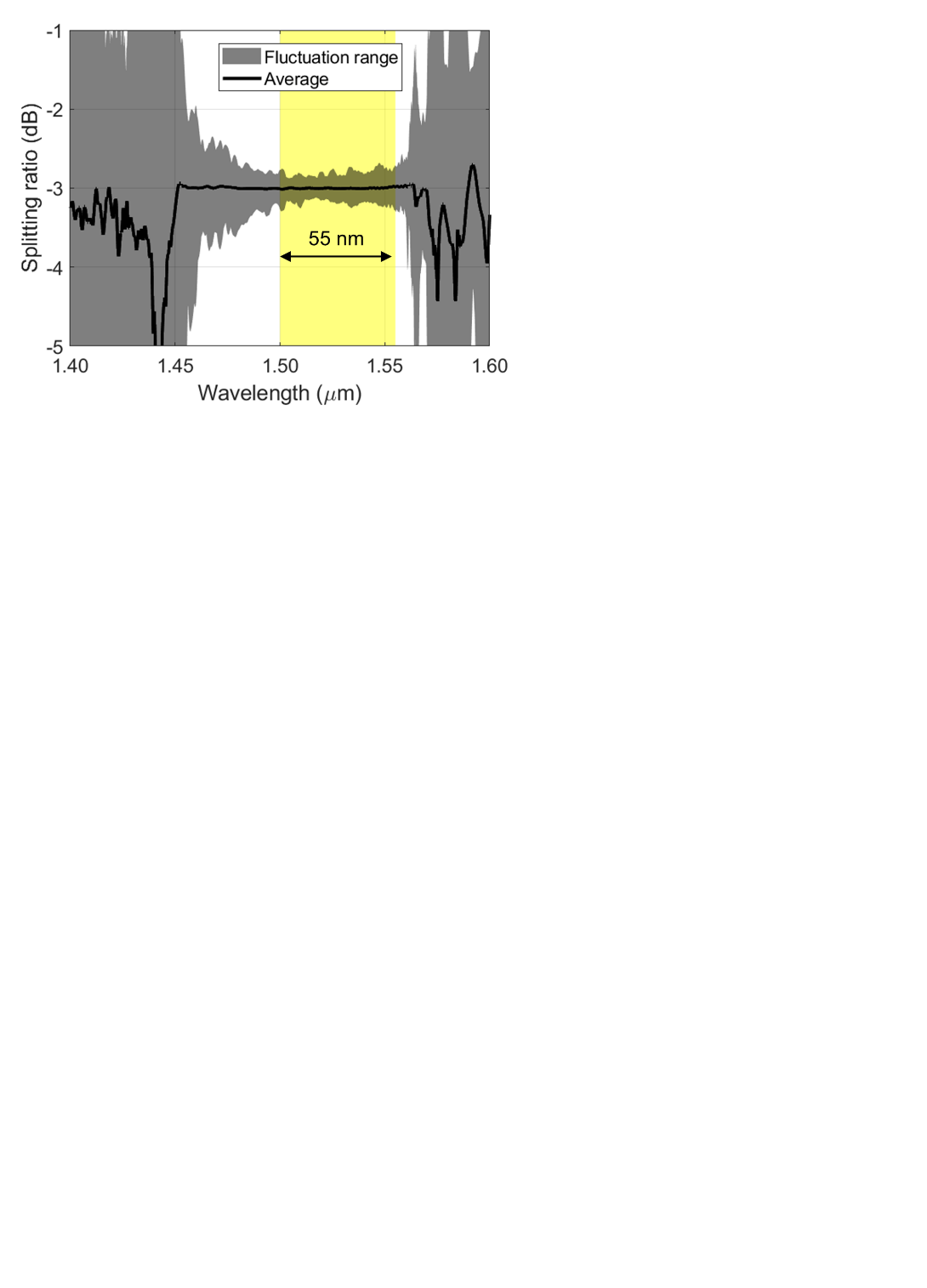


**Fig. S11 Robustness against non-uniform dimensional errors.** The splitting ratio of topological couplers with non-uniform dimensional errors. The gray region is the range between the minimum and maximum splitting ratio spectra. The solid line is the average of splitting ratio spectra. The gray region is within (3 ± 0.32) dB from 1500 nm to 1555 nm (yellow region). Simulations with random errors on different holes are performed for 100 times.

# Appendix E: Derivation and confirmation of interference process

In this section, we will derive the transmission of port1 and port2 when coherent light beams with phase difference of $\Delta\phi$ are input from port 3 and port 4, and confirm the analytical results with simulations.

Consider the input light of port 3 and port 4 have same intensity and a phase difference $\Delta\phi=\phi_{4}-\phi_{3}$. We can get the transmission of port1 and port4 by substituting $H_{3}=\frac{1}{\sqrt{2}}H_{0}$, $H_{4}=\frac{1}{\sqrt{2}}e^{i\Delta\phi}H_{0}$ into the scattering matrix expression to calculate the field amplitude

$H_{1}=\frac{1}{2}\left( 1+\exp i\Delta\phi\right)H_{0}$ (S21a)

$H_{2}=\frac{1}{2}\left( 1-\exp i\Delta\phi\right)H_{0}$ (S21b)

and the intensity

$\left| H_{1} \right|^{2}=\frac{1}{2}\left( 1+\cos\Delta\phi\right)\left| H_{0} \right|^{2}$ (S22a)

$\left| H_{2} \right|^{2}=\frac{1}{2}\left( 1-\cos\Delta\phi\right)\left| H_{0} \right|^{2}$ (S22b)

We can finally derive the transmissions $T_{i}=\frac{\left| H_{i} \right|^{2}}{\left| H_{0} \right|^{2}}$

$T_{1}=\cos^{2} \frac{\Delta\phi}{2}$ (S23a)

$T_{2}=\sin^{2} \frac{\Delta\phi}{2}$ (S23b)

To confirm the theoretical analysis, we perform the interference process in numerical simulations. As shown in Fig. S12a, two light beams input from port 3 and port 4 simultaneously, which are coherent with the same amplitude but have phase difference of $\Delta\phi$. Taking the wavelength of 1550 nm as an example, Fig. S12b plots the theoretically predicted (lines) and numerically simulated (dots) results of the relation between transmission and phase difference. The numerical results closely match the analytical predictions, confirming the accuracy and validity of our analysis.


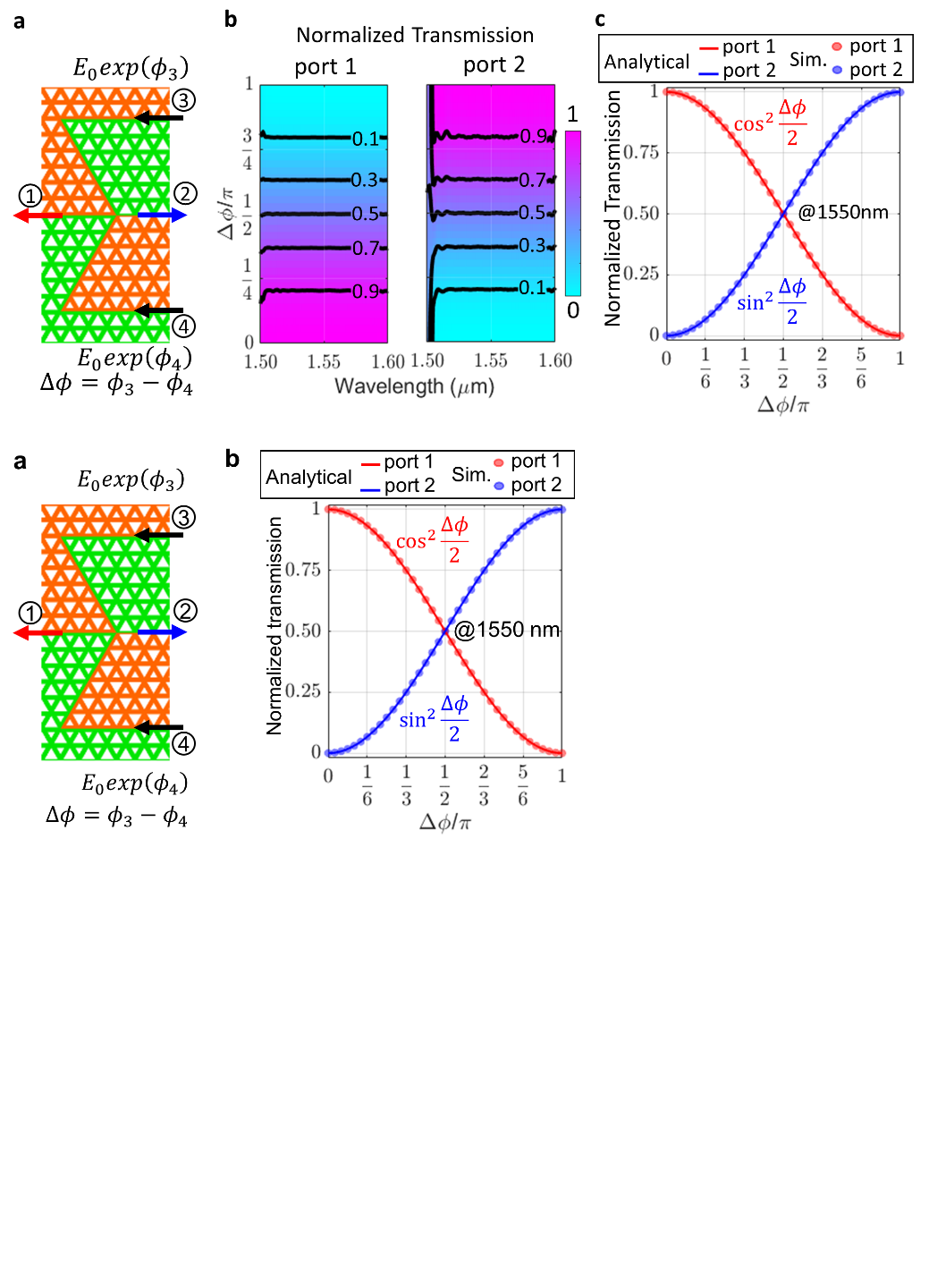


**Fig. S12 Analysis of interference process.** **a**, Propagation direction of light in the interference process. **b**, Analytical (solid line) and simulation (dot) results of the transmission of port 1 (red) and port 2 (blue) vary with phase difference $\Delta\phi$ at fixed wavelength 1550 nm.

# Appendix F: Data analysis

In this section, we describe the data processing methods utilized in simulation and experiment. The procedures for obtaining transmission and splitting ratio spectra will be introduced.


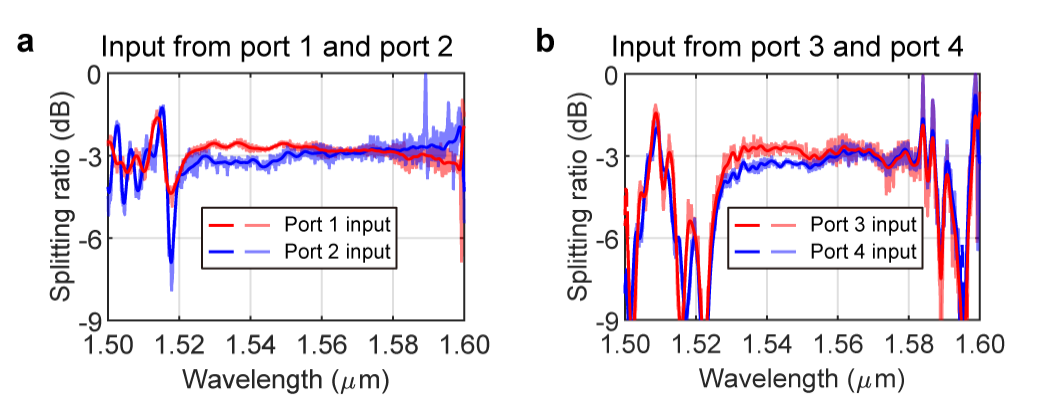


**Fig. S13 Filtering process of data.** Splitting ratio for the cases of inputting from (**a**) port 1 and port 2, (**b**) port 3 and port 4. The spectra before and after filtering process are plotted with semitransparent lines and opaque lines respectively.

To obtain the transmission of a device, the power data recorded by optical powermeter should be normalized by the power data for a reference sample. Here the reference sample is a strip waveguide. Note that the original transmission may arise the fringes due to the limited coupling efficiency of the grating coupler. To reduce the fringes, we apply a Gaussian filtering process to convolute the transmission spectra with a normalized Gaussian kernel. The standard deviation of the Gaussian kernel is set as σ = 0.5 nm. Figure S13 shows the splitting ratio spectra before and after the filtering process to illustrate the effect of the Gaussian filtering on the data.

In both simulations and experiments of topological coupler, we focus on the splitting phenomenon within the VPC structure. Therefore, it should exclude the effect of different coupling efficiencies between strip waveguides and the ports of VPCs. To do this, we use the transmission spectra of VPC waveguides as reference to normalize the transmission of couplers. For instance, when we input light from port 3 and analyze the transmission to port 1 and port 2, we utilize two samples of sharp-bent VPC waveguides as shown in Fig. S14. One of them contains only port 1 and port 3, while the other contains only port 2 and port 3. By using these samples as references, the output powers of port 1 and port 2 can be obtained by the normalized transmissions of *T*_13_ and *T*_23_. Furthermore, the splitting ratio of port 1 and port 2 can be calculated from the expressions of $\frac{T{}_{13}}{T{}_{13}+T{}_{23}}$ and $\frac{T{}_{23}}{T{}_{13}+T{}_{23}}$. In main text, we use the splitting ratio of port 1 to characterize the performance of couplers. One can easily get the splitting ratio of port 2 through the relation of $\frac{T{}_{13}}{T{}_{13}+T{}_{13}}+\frac{T{}_{23}}{T{}_{13}+T{}_{13}}=1$.


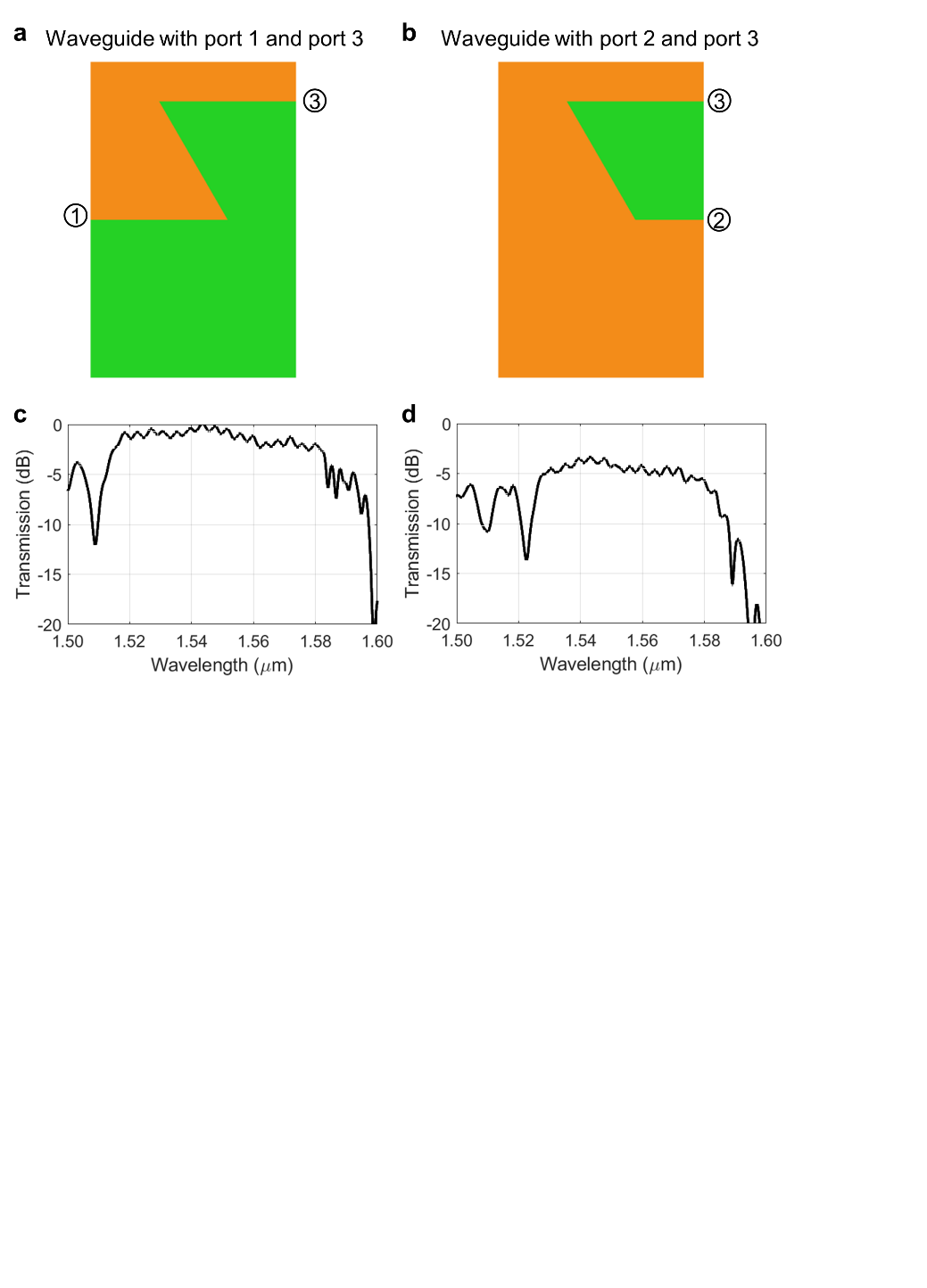


**Fig. S14 VPC waveguide samples for normalization. a** and **b**, Structures of harp-bent VPC waveguides with only (**a**) port 1 and port 3, and (**b**) port 2 and port 3 which are utilized in normalization. VPC1 and VPC2 are colored in orange and green respectively. **c** and **d**, Transmission spectra of two harp-bent waveguides. The transmission spectra represent the insertion loss for the cases of inputting or outputting at (**c**) port 1 and (**d**) port 2, respectively.

We can obtain the insertion loss of topological coupler from the transmission of the samples for normalization. From the transmission spectra, the insertion loss for all the input cases is less than 5.88 dB in 53 nm (1527 nm ~ 1580 nm). The difference of insertion loss between two input cases comes from the difference of the coupling structures between port 1 and port 2.

# Appendix G: The design and optimization of topological coupler

In this section, we describe the design and optimization procedure of topological photonic crystal and the topological coupler.

Firstly, we need to design the structure of VPCs. As the polarization of operation mode is TE-like mode, we choose the photonic crystal with holes in a slab [2]. Because of the requirement of *C*_3_ symmetry, VPCs usually have the graphene-like structure of honeycomb lattice and the shape of holes should have *C*_3_ symmetry, such as circle, triangle and hexagon. [3-5]. Here, we use triangular holes in this work. When the inversion symmetry is broken, the degeneration of photonic bands will be broken and a band gap emerges. In order to break the inversion symmetry, the sizes of holes in a unit cell are set to be different. As the materials and slab thickness are fixed, three structural parameters – lattice constant *a*, side-lengths of holes *s*_1_, *s*_2_ – are optimized to make the range of edge states in band gap cover the operation wavelength. We can change the middle wavelength of band gap by changing the lattice constant *a* and the summation of *s*_1_ and *s*_2_. We can expand the size of band gap by increasing the difference between *s*_1_ and *s*_2_. We note that in the above procedure, since the physical principle of the parameters’ effect on band structure is clear, the optimization direction is explicit. Besides, we only need to calculate the bulk band and the edge dispersions and do not need to perform the full-wave simulation of transmission process.

Then we design the structure of 3-dB coupler. Using the optimized structural parameters, we can confirm the robust transport performance and the operation range with a Z-shaped waveguide in transmission simulation. To reduce the insertion loss, we can optimize the coupling structure with a straight waveguide in simulation. In the design of 3-dB coupler, we need to follow the principle: the structure should be mirror-symmetric and there should not be unexpected transmission path. In this work, we utilize a structure with mirror symmetry and keep the distance between waveguides as $12\sqrt{3}a$ (9.145 μm) to prevent evanescent coupling of edge modes. Finally, we perform the transmission simulations to characterize the performance of couplers, such as the splitting ratio, bandwidth and tolerance.


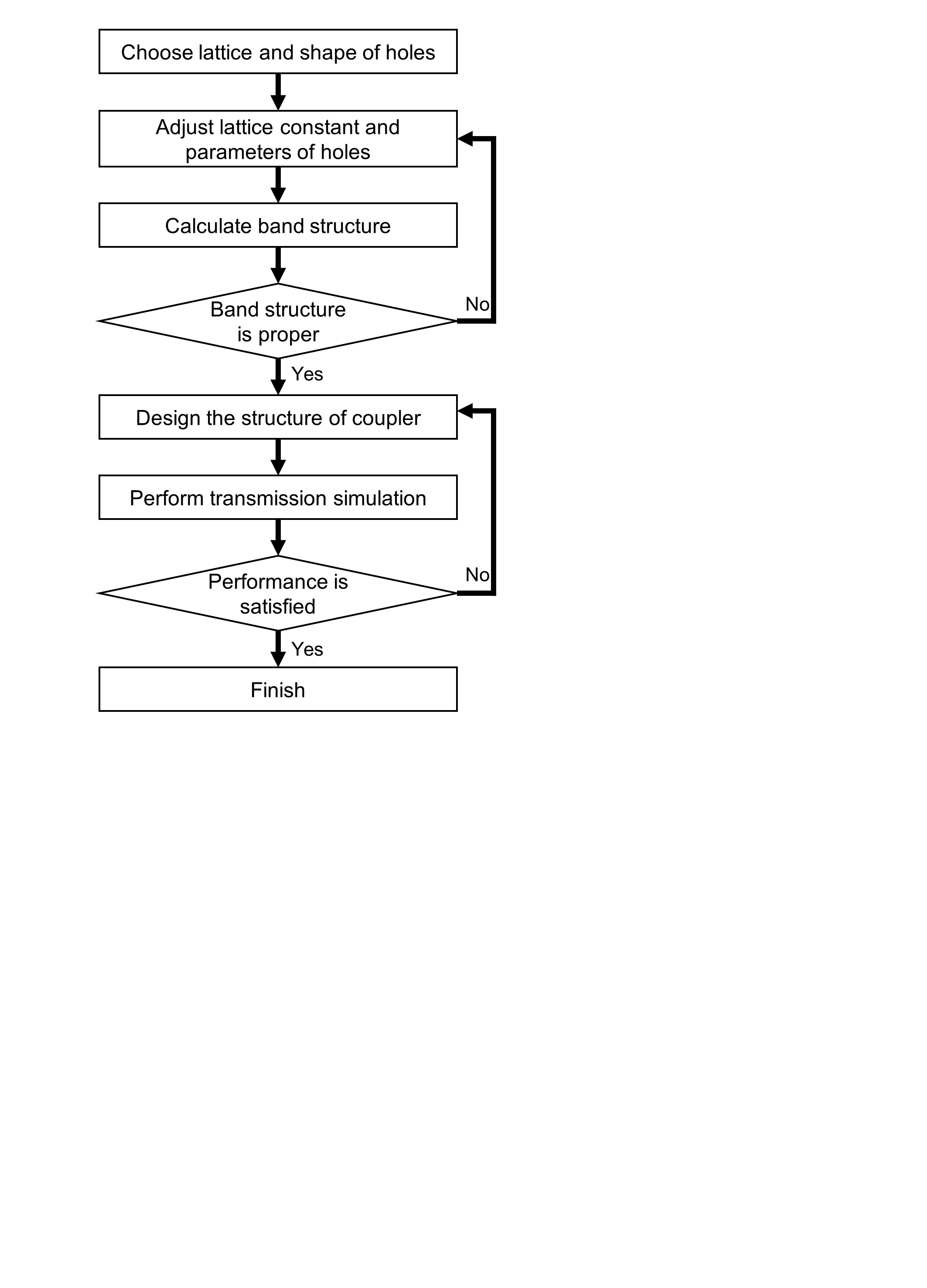


**Fig. S15 Design procedure of topological coupler.** The design procedure has two sections: the design of VPCs by solving eigenfrequency, and the design of coupler structure by transmission simulation.

The footprint of topological coupler can be further optimized. The size of topological coupler is limited by the transverse evanescent coupling of edge modes. To reduce the size of coupler, we need to increase the transverse decay speed of edge modes. According to Eq. (S3) - (S5), we can increase the transverse decay of edge modes by enlarging the band gap, which can be achieved by increasing the difference between *s*_1_ and *s*_2_. To enlarge the band gap as much as possible and fix the operation range, we remove the smaller hole and enlarge the larger hole as *s*_2_ = 0.82*a*. We note that the parameter is chosen by band structure calculation. Then we reduce the length of waveguides and the transverse distance between them. Compared with Fig. S8, the splitting ratio has no obvious change. The footprint of coupler is reduced from 23.46 μm × 27.44 μm to 15.54 μm × 13.72 μm.


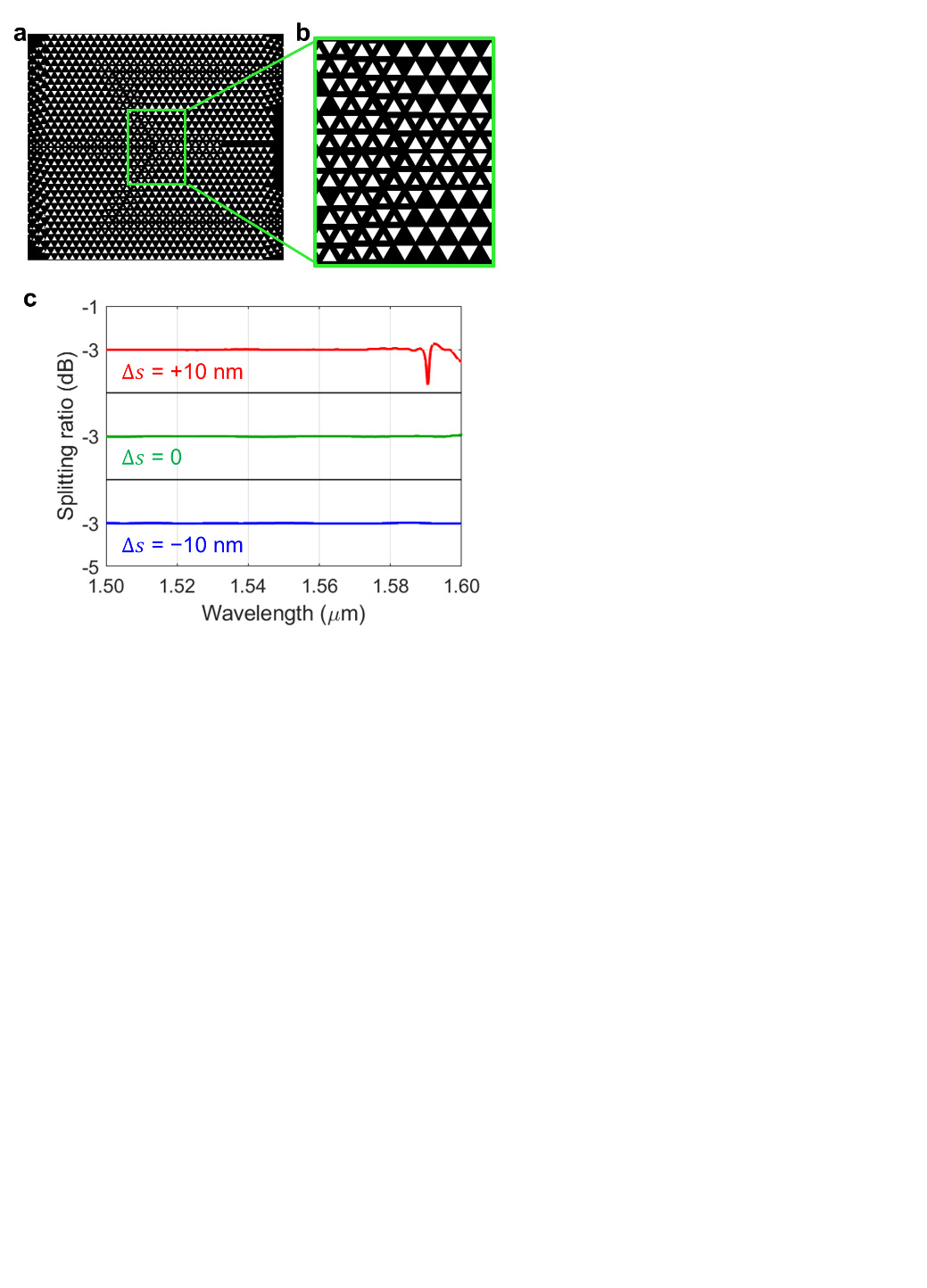


**Fig. S16 Optimized topological coupler with smaller size. a** and **b**, The (**a**) whole structure and the (**b**) zoomed structure details of optimized topological coupler. **c**, The splitting ratio spectra of optimized topological coupler when dimensional errors are introduced.

# Appendix H: Data processing procedure of distance measurement

In this section, we describe the data processing procedure details of on-chip distance measurement with topological interferometer.

Firstly, we translate the transmission spectra from $T\left( \lambda\right)$ into $T\left( \omega\right)$. Combining $T\left( \omega\right)$ and the ω-*k* relation of strip waveguides, we can obtain the $T\left( k \right)$ relation. By performing a Fourier transform on the $T\sim k$ relation, we obtain the $T\sim x$ relation. According to Eq. (S23), the transmission spectra is determined by the accumulated phase difference between two arms $T\left( \omega\right)=\frac{1}{2}T_{0}\left[ 1-\cos\Delta\phi\left( \omega\right) \right]$. The phase difference is determined by the length difference between two arms $\Delta\phi=k\Delta L$. The ideal $T\sim k$ relation has the form of $T\left( k \right)=\frac{1}{2}T_{0}\left[ 1-\cos\left( k\Delta L \right) \right]$. After the Fourier transform, there will be peaks at $x=0, \pm\Delta L$. We can extract the length difference between two arms from the peak positions of the Fourier transform result. When we have known the length of reference arm, we can derive the length of measurement arm.

We note that such interferometer not only can extract the length difference between silicon waveguides, but also has the function of measuring distance in free space based on the same principle. When we remove the DBR of measurement arm and terminate the waveguide with butt coupler, we can measure the distance in free space, which is the basic function of LiDAR. We can also construct the reference arm in the same method and use a mirror as reflector. The reflector of measurement arm is the structure to be detected. The measurement principle is same to the on-chip measurement process. As the dispersion of free space has linear form, we even do not need the dispersion of waveguides.

# Appendix I: Comparison of various 3-dB couplers

In this section, we compare our topological coupler with other reported 2×2 couplers. we mainly focus on 3-dB couplers and compare their performances including footprint, insertion loss, bandwidth, and tolerance in a table.

**Table S1 Comparison of various on-chip 2×2 couplers**

| **Ref.** | **Size (μm^2^)** | **IL (dB)** | **BW (nm)** | **Tolerance (Splitting ratio under error)** | **Design method** |
| --- | --- | --- | --- | --- | --- |
| [6] | 23.2 × 46.7 | < 0.23 | 135 | — | Inverse design |
| [7] | 18 × 31 | < 0.5 | 145 | — | Inverse design |
| [8] | 25 × 65 | < 0.74 | 100 | — | Inverse design |
| [9] | 15 × 35 | < 0.11 | 185 | (3 ± 0.7) dB in 185 nm, ±10 nm error (Exp.) | Inverse design |
| [10] | 5.25 × 17.25 | < 1 | 200 | (3 ± 0.4) dB in 300 nm, ±5 nm error (Sim.) | Inverse design |
| [11] | 1.3 × 1.3 | — | — | (4.8 ± 0.8) dB in 100 nm, ±10 nm error (Sim.) | Inverse design |
| [12] | 22.08×24.55 | < 3.65 | 24 | — | Topological principle |
| This work | 23.46 × 27.44 | < 5.88 | 53 | (3 ± 0.1) dB in 81 nm, ±10 nm error (Sim.)  (3 ± 0.6) dB in 48 nm, ±10 nm error (Exp.) | Topological principle |

IL: Insertion Loss; BW: Bandwidth.

As shown in Table S1, the topological coupler has achieved the same level of tolerance compared with other couplers. Although the bandwidth of topological coupler is smaller than others, the splitting ratio fluctuation is smaller than that of other couplers when similar dimensional errors are introduced. Therefore, topological coupler is suitable for the applications requiring small splitting ratio fluctuation.

Of course, inverse design can also be applied in the topological couplers. Inverse design can achieve the local optimum with algorithm, which is widely used to reach the extreme of performance. Topological design method and the inverse design are not incompatible. We can firstly design the device based on topological principle, and optimize the structure with inverse design. In this way, we can combine the benefit of topological principle on the explicit principle with clear optimization guidance and the advantage of inverse design on achieving local optimum.

Reference:

1. H. H. Li, "Refractive index of silicon and germanium and its wavelength and temperature derivatives," Journal of Physical and Chemical Reference Data **9**, 561-658 (1980).

2. J. D. Joannopoulos, S. G. Johnson, J. N. Winn, and R. D. Meade, *Photonic Crystals: Molding the Flow of Light,* 2 ed. (Princeton University Press, Princeton and Oxford, 2008).

3. X. T. He, E. T. Liang, J. J. Yuan, H. Y. Qiu, X. D. Chen, F. L. Zhao, and J. W. Dong, "A silicon-on-insulator slab for topological valley transport," Nature Communications **10**, 872 (2019).

4. M. I. Shalaev, W. Walasik, A. Tsukernik, Y. Xu, and N. M. Litchinitser, "Robust topologically protected transport in photonic crystals at telecommunication wavelengths," Nature Nanotechnology **14**, 31-34 (2019).

5. Y. Chen, X. T. He, Y. J. Cheng, H. Y. Qiu, L. T. Feng, M. Zhang, D. X. Dai, G. C. Guo, J. W. Dong, and X. F. Ren, "Topologically Protected Valley-Dependent Quantum Photonic Circuits," Physical Review Letters **126**, 230503 (2021).

6. I. K. Kim, D. U. Kim, V. H. Nguyen, S. Han, and T. J. Seok, "High-Performance and Compact Silicon Photonic 3-dB Adiabatic Coupler Based on Shortest Mode Transformer Method," Ieee Photonics Journal **13**, 6601106 (2021).

7. J. M. F. Cabanillas, B. Zhang, M. A. Popovic, and Ieee, "Demonstration of 3+0.12 dB power splitting over 145 nm optical bandwidth in a 31-μm long 3-dB rapid adiabatic coupler," in *Optical Fiber Communications Conference and Exposition (OFC)*, 2020), paper Th1A.2.

8. L. H. Xu, Y. Wang, A. Kumar, E. El-Fiky, D. Mao, H. Tamazin, M. Jacques, Z. P. Xing, G. Saber, and D. V. Plant, "Compact high-performance adiabatic 3-dB coupler enabled by subwavelength grating slot in the silicon-on-insulator platform," Optics Express **26**, 29873-29885 (2018).

9. H. Yun, L. Chrostowski, and N. A. F. Jaeger, "Ultra-broadband 2 x 2 adiabatic 3 dB coupler using subwavelength-grating-assisted silicon-on-insulator strip waveguides," Optics Letters **43**, 1935-1938 (2018).

10. C. C. Ye and D. X. Dai, "Ultra-Compact Broadband 2 x 2 3 dB Power Splitter Using a Subwavelength-Grating-Assisted Asymmetric Directional Coupler," Journal of Lightwave Technology **38**, 2370-2375 (2020).

11. L. He, D. N. Liu, J. X. Gao, W. X. Zhang, H. Z. Zhang, X. Feng, Y. D. Huang, K. Y. Cui, F. Liu, W. Zhang, and X. D. Zhang, "Super-compact universal quantum logic gates with inverse-designed elements," Science Advances **9**, eadg6685 (2023).

12. H. W. Wang, L. Sun, Y. He, G. J. Tang, S. H. An, Z. Wang, Y. H. Du, Y. Zhang, L. Q. Yuan, X. T. He, J. W. Dong, and Y. K. Su, "Asymmetric Topological Valley Edge States on Silicon-On-Insulator Platform," Laser & Photonics Reviews **16**, 2100631 (2022).
